# Sb-Doped SnO_2_ Hollow Spheres for Low-Resistance and Highly Selective Xylene Sensors

**DOI:** 10.3390/nano16050313

**Published:** 2026-02-28

**Authors:** Jung-Hoo Seo, Seong-Young Yoon, Sang-Myeong Lee, Seong-Yong Jeong

**Affiliations:** Division of Advanced Materials Engineering, Kongju National University, Cheonan 31080, Republic of Korea

**Keywords:** Sb-SnO_2_, xylene, hollow spheres, selectivity, low electrical resistance, ultrasonic spray pyrolysis

## Abstract

It is important to be able to detect xylene with high selectivity and low sensor resistance when monitoring indoor and outdoor air quality. In this study, we report the development of Sb-doped SnO_2_ hollow spheres synthesized via ultrasonic spray pyrolysis for high-performance xylene detection with significantly reduced sensor resistance. The 2 mol% Sb-doped SnO_2_ sensor exhibited a remarkably high response (*S*_X_ = 24.0) and selectivity (*S*_X_/*S*_E_ = 3.4) toward 5 ppm xylene at 300 °C. Notably, the sensor resistance in air (*R*_a_) was reduced by ~200-fold compared to that of pure SnO_2_, reaching a practical level of 38.5 kΩ, which enables cost-effective signal measurement. Furthermore, the 2Sb-SnO_2_ sensor demonstrated a low detection limit of 50 ppb and rapid response times (4–5 s). These results suggest that Sb doping is a highly effective strategy for engineering low-resistance and highly selective SnO_2_ gas sensors. This study could pave the way for a practical approach to designing xylene detection systems for indoor air monitoring.

## 1. Introduction

Xylene is a harmful and ubiquitous indoor pollutant among volatile organic compounds (VOCs), which can cause various adverse health effects on the central nervous system, respiratory, kidney, lung, and heart [1,2,3,4]. Xylene is known as a representative aromatic hydrocarbon widely used as a solvent and raw material in various industries, such as paints, printing inks, adhesives, and cigarette smoke. However, highly selective and sensitive detection of xylene is challenging due to its high molecular stability [5,6,7,8,9,10,11,12].

Generally, conventional analytical instruments such as gas chromatography-mass spectrometry (GC-MS) [13] and fourier transform infrared spectroscopy (FTIR) [14] are widely used for harmful xylene detection. However, these methods are bulky and expensive, thereby limiting real-time and on-site xylene detection. Therefore, metal oxide semiconductor-based gas sensors have attraction as a promising alternative due to low cost, simple structure, easy miniaturization, fast response, and high sensitivity [15,16,17,18,19,20,21]. However, metal oxide semiconductor-based gas sensors suffer from poor selectivity because of their simple sensing mechanism between oxide surface and gases. The sensing mechanism of n-type sensors is generally governed by surface adsorption of oxygen species (O_2_^−^, O^−^), which extract electrons from the conduction band and form a surface depletion layer. Upon exposure to reducing gases such as xylene, the adsorbed oxygen species react with the gas molecules and release trapped electrons back to the semiconductor, thereby modulating the surface potential barrier and depletion width. The resulting change in charge carrier concentration near the surface leads to a measurable variation in electrical resistance. Therefore, gas response in oxide chemiresistors is primarily determined by modulation of the surface depletion layer.

Considerable efforts have been devoted to improving xylene selectivity by developing nanostructures and doping/loading of catalysts [22,23,24]. Although these nanostructures and catalyst designs can enhance selectivity to the target gas, the sensor resistance (*R*_a_) often increases to an immeasurable level (over MΩ range) [1,25,26,27]. For example, Kim et al. [28] reported that Nb-doped NiO hollow spheres exhibited an ultrahigh response towards methylbenzene. However, the *R*_a_ values substantially increased (1800 times) with Nb doping at 350 °C. The same group further suggested that Cr doping of the NiO hierarchical nanostructures significantly increased sensor resistance [29]. Maebana et al. [30] also reported that adding CuO to ZnO remarkably increased the xylene response, accompanied by an increase in the *R*_a_ value. High *R*_a_ values can hinder the cost-effective detection of xylene. Accordingly, highly selective and sensitive xylene detection without increased *R*_a_ values is important for practical use.

Among metal oxide semiconductors, SnO_2_ has been extensively investigated for chemiresistive gas sensing due to its robust chemical stability and high gas response. In particular, SnO_2_ nanostructures and dopant/catalyst strategies have been widely explored to tune both selectivity and electrical resistance. Motivated by the selectivity–resistance trade-off discussed above, we investigated Sb-doped SnO_2_ hollow spheres using ultrasonic spray pyrolysis. The Sb doping concentration was systematically varied at 2, 5, and 10 mol% to investigate its influence on both the electrical resistance and gas-sensing characteristics. Our results demonstrate that 2 mol% Sb-doped SnO_2_ hollow spheres successfully addressed the high-resistance issue, exhibiting an *R*_a_ value ~200 times lower than that of pure SnO_2_. At the optimal operating temperature of 300 °C, the 2Sb-SnO_2_ sensor showed a high response (*S*_X_ = 24.0) and superior selectivity toward 5 ppm xylene, with a low detection limit of 50 ppb. The drastic reduction in resistance and the enhanced sensing performance are discussed in relation to the charge compensation mechanism arising from the substitution of Sb^5+^ into Sn^4+^ lattice sites and the unique hollow morphology. This study provides a practical and effective strategy for designing low-resistance SnO_2_ sensors capable of highly sensitive and selective xylene detection for indoor air quality monitoring.

## 2. Experimental

### 2.1. Synthesis of Sensing Materials

Pure and Sb-SnO_2_ hollow spheres were synthesized by ultrasonic spray pyrolysis (Figure S1a). For pure SnO_2_ hollow spheres, Tin (ll) chloride dihydrate (0.1 M, 2.2565 g, SnCl_2_·2H_2_O, 99.99%, Sigma-Aldrich, St. Louis, MO, USA), citric acid monohydrate (0.25 M, 5.2535 g, C_6_H_8_O_7_·H_2_O, 99.5%, Samchun, Pyeongtaek, Republic of Korea), and a diluted hydrochloric acid solution (35.0–37.0%, HCl: distilled water = 1:99 by vol%) (1 mL) were dissolved in 99 mL distilled water for preparation of a spray solution. After stirring for 1 h at room temperature for homogeneous mixing, the precursor solution was placed into an ultrasonic atomizer. Droplets were generated via an ultrasonic nebulizer (resonant frequency: 1.7 MHz) and transported into a quartz tube (inner diameter = 50 mm, length = 1200 mm) in a high-temperature furnace heated to 700 °C using a carrier gas (air, 10 L/min). For Sb-SnO_2_ hollow spheres, antimony (III) chloride (SbCl_3_, 99.00%, Sigma-Aldrich, USA) was added to the spray solution at concentrations of [Sb]/[Sn + Sb] × 100 of 2, 5 and 10 mol%. The precursor powders were collected in a Teflon bag filter and subsequently annealed at 600 °C for 3 h. The morphology of the hollow spheres was analyzed by field-emission scanning electron microscopy (FE-SEM, MIRA3-LMH, Tescan, Brno, Czech Republic). The crystal structure and phase of pure and Sb-doped SnO_2_ were analyzed by X-ray diffractometer (XRD, D8 Advance, Bruker AXS GmbH, Karlsruhe, Germany) and X-Ray Photoelectron Spectrometer (XPS, K-Alpha, Thermo Fisher Scientific, Waltham, MA, USA).

### 2.2. Fabrication of Gas Sensor

Pure and Sb-doped SnO_2_ hollow spheres were mixed with a terpineol-based organic ink vehicle (FCM, Lewis Center, OH, USA) as an organic binder to prepare a slurry (powder:binder = 1:4 by weight). The gas sensors were fabricated by screen-printing the slurry onto the alumina substrate (area: 1.5 × 1.5 mm^2^) with two patterned Au electrodes on the top and a micro heater on the bottom. Direct thickness quantification (e.g., cross-sectional imaging) was not performed in this study; all sensors were prepared using an identical printing protocol to minimize sample-to-sample variation. The sensors were heat treated at 450 °C for 2 h to remove organic impurities and water. Herein, the Sb-doped SnO_2_ hollow spheres are referred as *x*Sb-SnO_2_ (*x* = 2, 5, and 10) for simplicity.

### 2.3. Gas Sensing Measurement

The sensors were placed in a homemade sensing chamber. Prior to measurement of the gas sensing characteristics, all sensors were heated at 450 °C for 1 h in dry air for stabilization. The gas atmosphere was controlled using mass flow controllers and an automatic four-way valve at a constant total flow rate of 200 cm^3^ min^−1^. The concentrations (5 ppm) of analyte gases (xylene, ethanol, acetone, formaldehyde, ammonia, and propane) were precisely regulated by controlling the mixing ratios between synthetic gas and dry air. Prior to each sensing measurement, the test chamber was thoroughly purged with dry air. Xylene concentration was confirmed by Proton Transfer Reaction Time-of-Flight Mass Spectrometry (PTR-TOF-MS, PTR-ToF 1000, IONICON, Innsbruck, Austria). The two-probe DC resistance of the sensors was measured using a multimeter (Model 3706A, Keithley Instruments, Cleveland, OH, USA), with data acquisition handled by a computer. A constant DC bias voltage of 1 V was applied across the Au electrodes during resistance measurements (two-probe DC configuration), and the resistance data were recorded at a sampling interval of 1 s.

## 3. Results and Discussion

**Figure 1:**
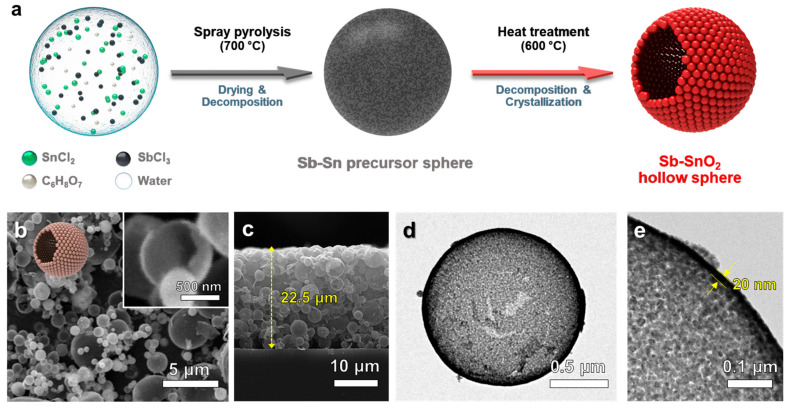
(**a**) Schematic illustration of the synthesis procedure. (**b**) Scanning electron microscopy (SEM), (**c**) cross-sectional SEM, (**d**) transmission electron microscopy (TEM), and (**e**) high-resolution TEM images of pure SnO_2_ spheres.

**Figure 2:**
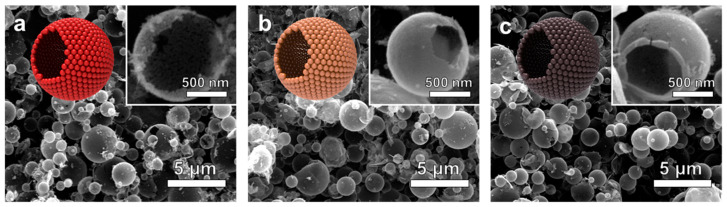
SEM images of the (**a**) 2Sb-, (**b**) 5Sb-, and (**c**) 10Sb-doped SnO_2_ sensors.

Pure SnO_2_ and *x*Sb-SnO_2_ (*x* = 2, 5, and 10) spheres were prepared using ultrasonic spray pyrolysis (Figure 1a). The spherical structures of the pure SnO_2_ and *x*Sb-SnO_2_ were confirmed through SEM analysis (Figure 1 and Figure 2). The average diameter of Pure SnO_2_ and 2Sb-SnO_2_ spheres was 1.13 ± 0.82 μm and 1.11 ± 0.52 μm, respectively (Figure S2). The hollow morphology was confirmed through the broken shell of the powders (Inset of Figure 1b). In addition, Figure 1d shows bright contrast in the central region of the SnO_2_ sphere, in contrast to the dark contour observed in the outer region. The shells exhibited a thickness of ~20 nm, and each sphere consisted of numerous primary particles with sizes of ~12 nm (Figure 1e).

**Figure 3:**
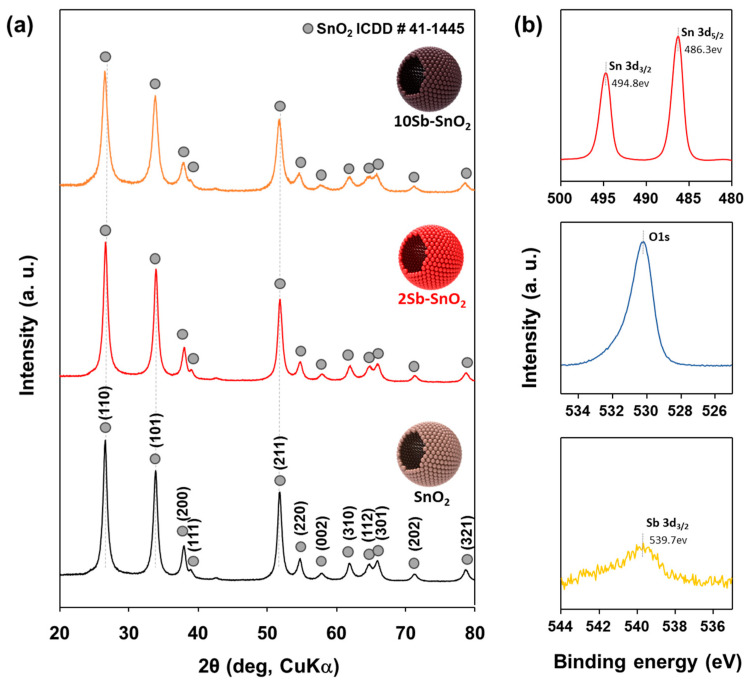
(**a**) XRD pattern of pure SnO_2_, 2Sb-SnO_2_, and 10Sb-SnO_2_ sensors. (**b**) X-ray photoelectron spectroscopy (XPS) spectra of the 2Sb-SnO_2_ sensor.

The phase and crystallinity of the resulting powders were analyzed by X-ray diffraction (XRD) using CuKα radiation (Figure 3a). The tetragonal SnO_2_ phase (ICDD #41-1445) was identified in the pure SnO_2_ and Sb-SnO_2_ hollow spheres through XRD analysis. The crystallite sizes of the SnO_2_ phase for the 2Sb-SnO_2_ and 10Sb-SnO_2_ spheres were estimated to be 11.5 ± 1.7 and 9.8 ± 1.1 nm, respectively, which are smaller than that of pure SnO_2_ spheres (12.1 ± 0.8 nm). This trend suggests that Sb doping tends to suppress crystallite growth during the synthesis process. In addition, the fact that only SnO_2_ peaks were observed, with no secondary phases even after antimony addition, indicates a successful solid solution of Sb within the SnO_2_ lattice. The absence of detectable secondary phases in XRD, even at higher Sb concentrations, suggests that Sb is homogeneously incorporated into the SnO_2_ lattice rather than forming segregated Sb-rich domains. Considering the relatively low doping levels (2–10 mol%), phase segregation is unlikely to be within the detection limit of XRD. The surface chemical states of the 2Sb-SnO_2_ sample were examined by X-ray photoelectron spectroscopy (XPS) (Figure 3b and Figure S3). The XPS spectra were modified by the C 1s peak at a binding energy of 284.6 eV. The Sn 3d spectrum displays two characteristic peaks at approximately 486.3 eV and 494.8 eV, corresponding to Sn 3d_5/2_ and Sn 3d_3/2_, respectively, indicating that Sn is predominantly present in the Sn^4+^ oxidation state. A weak but discernible Sb-related signal is observed in the Sb 3d region, suggesting the presence of Sb species at the surface of the 2Sb-SnO_2_ sample.

**Figure 4:**
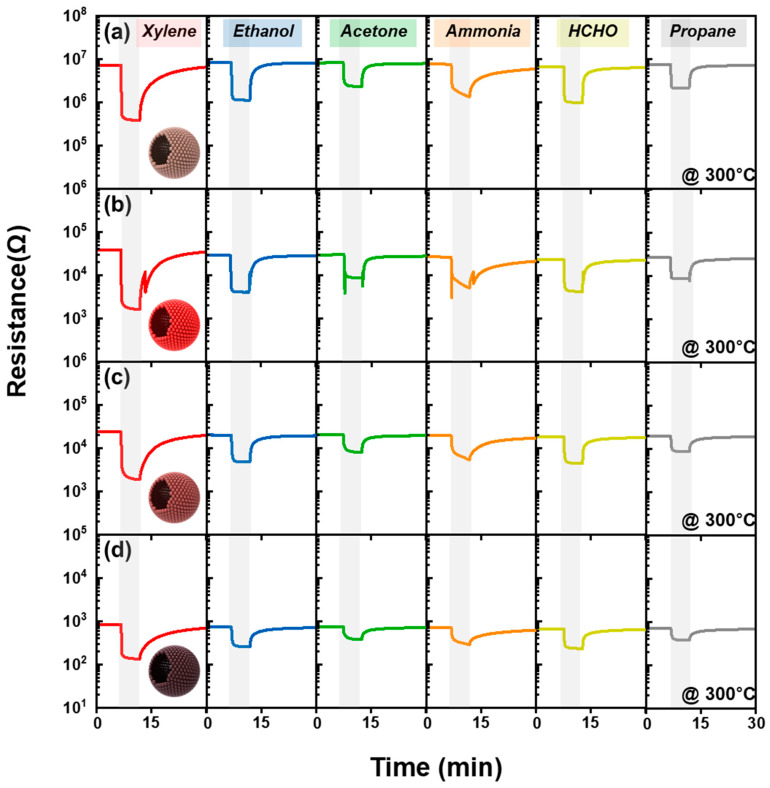
Dynamic sensing transients of (**a**) pure SnO_2_, (**b**) 2Sb-SnO_2_, (**c**) 5Sb-SnO_2_, and (**d**) 10Sb-SnO_2_ sensors at 300 °C.

**Figure 5:**
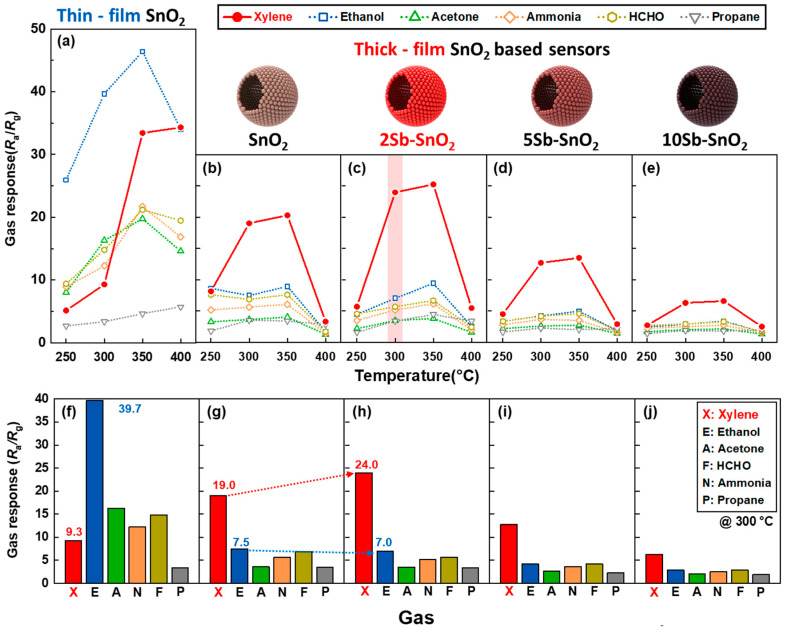
(**a**–**e**) Gas-sensing characteristics (at 250–400 °C) and (**f**–**j**) gas response (at 300 °C) to 5 ppm of analytes of pure SnO_2_ and Sb-doped SnO_2_ sensors.

The gas sensing characteristics of pure SnO_2_ and Sb-SnO_2_ sensors were investigated toward 5 ppm of xylene (X), ethanol (E), acetone (A), formaldehyde (F), ammonia (N), and propane (P) at 250–400 °C (Figure 4 and Figure 5). All sensors exhibited typical n-type semiconductor behavior where resistance drops in reducing gases and returns to the initial state in air (Figure 4) [15,31,32,33]. Accordingly, the gas response (*S*) was defined as

*S* = *R*_a_/*R*_g_ (1)

Initially, the thin-film SnO_2_ sensor (powder:binder = 1:6 by weight) exhibited a high response to ethanol at 250–350 °C (Figure 5a,f). This is consistent with the typical sensing characteristics of pure SnO_2_ sensors, where selective detection of xylene is difficult due to the cross-response to ethanol [22,23,34,35,36,37].

**Figure 6:**
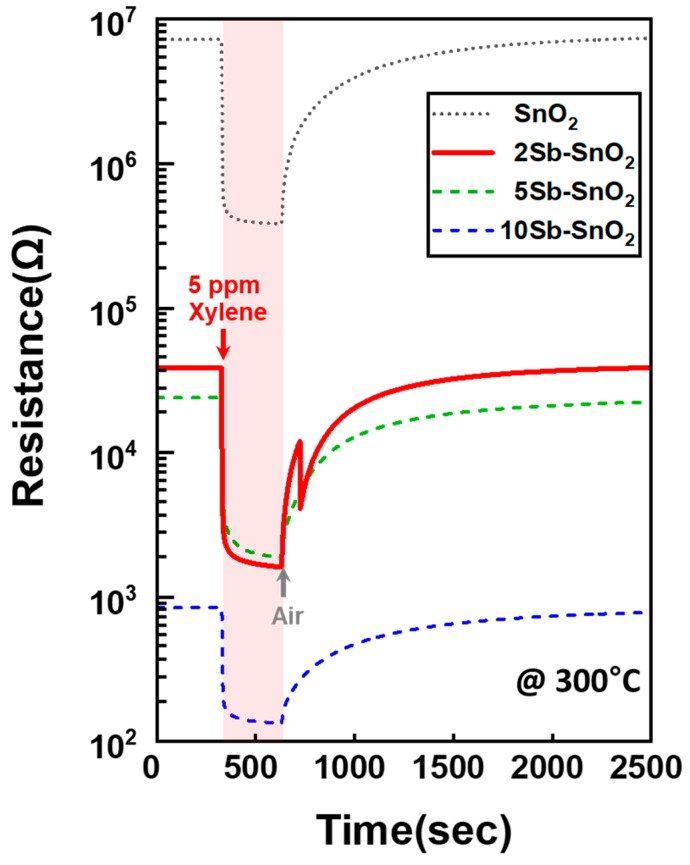
Sensor resistance (*R*_a_) of pure and Sb-doped SnO_2_ sensors at 300 °C.

However, the thick-film SnO_2_ sensor showed a significantly higher response to xylene than to other interfering gases, including ethanol (Figure 5b). The enhanced xylene selectivity can be attributed to oxidation of the highly reactive interfering gases in the gas-sensing process. Interestingly, the high gas response to xylene at 300 °C was probably obtained due to partial oxidation into highly reactive forms (i.e., reforming) (Figure 5g). Nevertheless, the resistance in air (*R*_a_) was notably high (7223.8 kΩ at 300 °C), which could hamper cost-effective resistance measurement in practical applications (Figure 6, Table 1, and Note S1).

Similar xylene sensing characteristics were maintained after addition of Sb catalyst to SnO_2_. For example, the response order (xylene > interfering gases) of Sb-doped SnO_2_ sensors at 300 and 350 °C was consistent with that of the thick-film pure SnO_2_ sensor (Figure 5b–e,g–j and Figure S4). The 2Sb-SnO_2_ sensor showed a comparable response to xylene and interfering gases (*S*_Xylene_ ≈ *S*_Interferent_) at 250 °C. It should be noted that the xylene response significantly increased from 19.0 (pure SnO_2_) to 24.0 (2Sb-SnO_2_) with a negligibly low gas response to the interfering gases at 300 °C (Figure 5c). For example, the 2Sb-SnO_2_ sensor exhibited a remarkably high xylene response (*S*_X_ = 24.0) compared to 5ppm ethanol (*S*_E_ = 7.0), acetone (*S*_A_ = 3.5), ammonia (*S*_N_ = 5.2), HCHO (*S*_F_ = 5.7), propane (*S*_P_ = 3.4) at 300 °C. Although the 1Sb-SnO_2_ sensor exhibited a higher response (*S*_X_ = 25.7 at 300 °C) to xylene than 2Sb-SnO_2_ (*S*_X_ = 24.0 at 300 °C), its selectivity (*S*_X_/*S*_E_ = 3.2) towards xylene was lower than that of 2Sb-SnO_2_ (*S*_X_/*S*_E_ = 3.4). Moreover, it was noted that the *R*_a_ value of the 2Sb-SnO_2_ sensor was remarkably reduced to 38.5 kΩ at 300 °C (Figure 6 and Table 1). Higher Sb doping further decreased the *R*_a_ value in the 5Sb- and 10Sb-SnO_2_ sensors. Although selective xylene sensing characteristics were maintained in the 5Sb-SnO_2_ and 10Sb-SnO_2_ sensors, the responses to all analyte gases drastically decreased across the entire sensing temperature range (250–400 °C) when increasing the Sb doping concentration to 5 and 10 mol% (Figure 5d,e). The above results indicate that Sb-doped SnO_2_ hollow spheres could be an excellent candidate for highly selective xylene detection with low *R*_a_ values.

**Figure 7:**
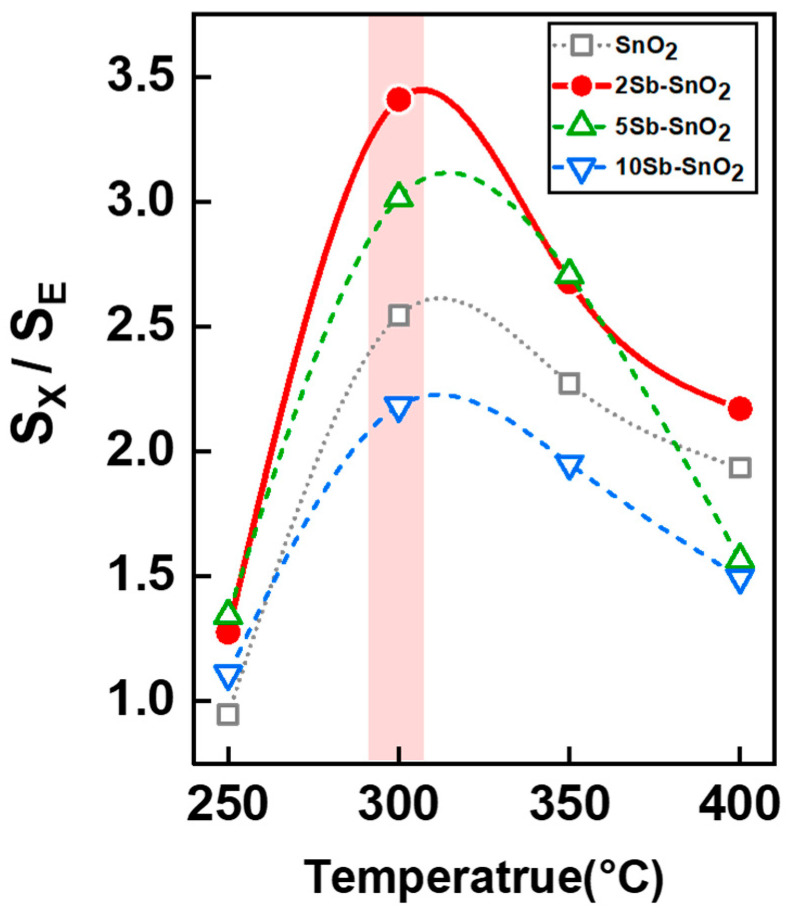
Selective xylene detection (*S*_Xylene_/*S*_Ethanol_) properties of pure SnO_2_ and Sb-doped SnO_2_ sensors.

The xylene selectivity (*S*_X_/*S*_E_) was defined as the response ratio of xylene to the major interferant ethanol [38,39,40,41,42] and plotted as a function of operating temperature (250–400 °C) (Figure 7 and Note S2). The xylene selectivity (*S*_X_/*S*_E_) of the pure SnO_2_ sensor ranged from 0.9 to 2.5 at 250–400 °C, whic increased to 1.2–3.2 upon 2 mol% Sb doping. However, the *S*_X_/*S*_E_ value gradually decreased with further Sb doping to 5 and 10 mol%. Note that all sensors exhibited the highest *S*_X_/*S*_E_ values at 300 °C with corresponding values of 2.5, 3.4, 3.0, and 2.1 for pure SnO_2_, 2Sb-, 5Sb-, and 10Sb-SnO_2_ sensors, respectively. Considering both selectivity (*S*_X_/*S*_E_) and response (*S*_X_) of the sensors, 2Sb-SnO_2_ hollow spheres operating at 300 °C were determined to be the most suitable for highly sensitive and selective detection of xylene.

For sensor applications, low sensor resistance is also an important factor [43,44,45,46]. As described earlier, the pure SnO_2_ sensor exhibited a high *R*_a_ value of 7223.8 kΩ at 300 °C. This high resistance of sensing materials can hinder cost-effective resistance measurements using conventional electric circuits for actual applications. Therefore, it is important to reduce sensor resistance to below the MΩ level. In contrast, the *R*_a_ values of the *x*Sb-SnO_2_ (*x* = 2, 5, and 10) sensors drastically decreased with increased Sb doping concentration (Figure 6 and Table 1). For example, the low *R*_a_ values for the 2Sb-, 5Sb-, and 10Sb-SnO_2_ sensors were achieved to 38.5 kΩ, 23.9 kΩ, and 0.8 kΩ at 300 °C, respectively. It was noted that these values were 188 (2Sb-SnO_2_), 302 (5Sb-SnO_2_), and 9028 (10Sb-SnO_2_) times lower than that of pure SnO_2_ (7223.8 kΩ).

The drastic decrease in the resistance with Sb doping is understood as a charge-compensation mechanism during the substitution of host Sn^4+^ with dopant Sb^5+^ within the lattice. Using Kröger–Vink notation, the defect chemical equation can be described as follows:

(2) $${Sb}_{2}O_{5}\overset{{2SnO}_{2}}{\to }2Sb\begin{array}{c} \cdot \\ Sn \end{array}+4O\begin{array}{c} X\\ O \end{array}+\frac{1}{2}O_{2}\left(g\right)+2e^{-}$$

According to this equation, the substitution of Sn^4+^ with Sb^5+^ generates excess electrons to maintain electroneutrality. This donor-type substitution increases the background electron concentration in the n-type SnO_2_ matrix. The XPS results (Figure 3b), showing Sb species consistent with a pentavalent state while preserving the Sn^4+^ framework, support this substitutional donor-doping mechanism. Accordingly, the resistance systematically decreases with increasing Sb content (2, 5, and 10 mol%). Importantly, despite this substantial reduction in *R*_a_ values, the selective xylene sensing characteristics of SnO_2_ are maintained (Figure 5b–e and Figure 6, Table 1), indicating that carrier modulation improves electrical conductivity without fundamentally altering the surface reaction selectivity. Although Sb doping increases the baseline carrier concentration, the sensing response is not governed solely by a simple dilution effect based on absolute carrier change. In chemiresistive sensors, the gas response is primarily determined by modulation of the surface depletion layer and the associated potential barrier. At moderate Sb doping (2 mol%), the carrier concentration increases while the depletion layer remains sufficiently developed to allow effective resistance modulation upon xylene adsorption. In contrast, excessive Sb doping (5 and 10 mol%) significantly narrows the depletion region, thereby reducing the relative barrier modulation and suppressing the response (Figure 5d,e). Therefore, the enhanced response observed for 2Sb-SnO_2_ can be understood as arising from an optimal balance between baseline conductivity and depletion-layer modulation (Figure 5c,h).

The enhanced xylene response and selectivity observed in 2Sb-SnO_2_ may be attributed to the catalytic effect of Sb. However, the 5Sb- and 10Sb-SnO_2_ sensors showed low gas responses to all analyte gases. For instance, the reduced response of 5Sb- and 10Sb-SnO_2_ sensors can be attributed to the increased background charge carrier concentration and the reduced adsorption of oxygen ions caused by excessive Sb addition. Generally, when a fixed amount of charge is injected into n-type oxide chemiresistive sensors, a lower background charge carrier concentration leads to higher variation of resistance (response, *R*_a_/*R*_g_). Therefore, the response of 5Sb- and 10Sb-SnO_2_ sensors is drastically decreased by the excessive increase in background charge carrier concentration caused by elevated Sb doping levels (5 and 10 mol%). Furthermore, the response is suppressed by the reduction in oxygen adsorption sites upon Sb doping, as Sb atoms occupy SnO_2_ surface sites and inhibit surface reactions. Consequently, 2 mol% Sb is the most efficient doping concentration for practical SnO_2_-based sensors, providing low electrical resistance and high xylene response.

**Figure 8:**
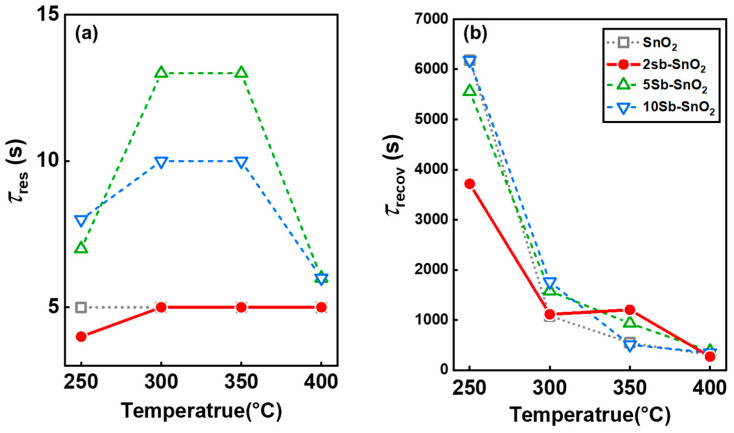
90% response (*τ*_res_) and recovery (*τ*_recov_) time of (**a**) pure SnO_2_ and (**b**) Sb-doped SnO_2_ sensors at temperatures in the range of 250–400 °C.

The response and recovery times were defined as the time required to reach 90% of the resistance variation upon exposure to xylene and air, respectively (Figure 8). Both pure SnO_2_ and 2Sb-SnO_2_ sensors exhibited rapid response times (*τ*_res_ = 4–5 s) across the entire temperature range (250–400 °C), whereas the 5Sb-SnO_2_ and 10Sb-SnO_2_ sensors showed slow response times (*τ*_res_ = 6–13 s and 6–10 s, respectively) (Figure 8a). In contrast, all sensors exhibited sluggish recovery times toward xylene (*τ*_recov_= 269–6179 s) at 250–400 °C (Figure 8b). The *τ*_recov_ values of pure SnO_2_ (1081 s) and 2Sb-SnO_2_ (1113 s) were remarkably lower than those of 5Sb-SnO_2_ (1577 s) and 10Sb-SnO_2_ (1760 s) at the optimal sensing temperature of 300 °C.

**Figure 9:**
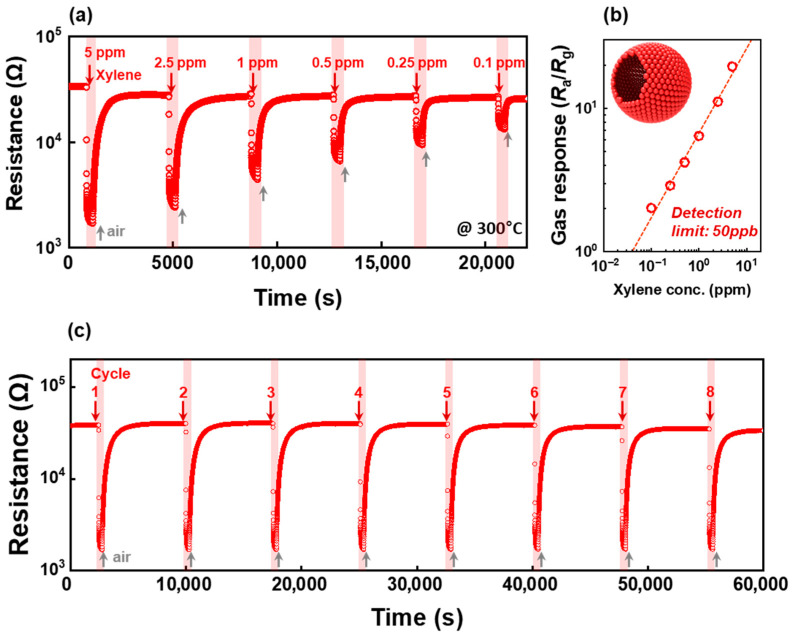
(**a**) Dynamic sensing transient of the 2Sb-doped SnO_2_ sensor to 5–0.1 ppm of xylene and (**b**) cyclic responses (*R*_a_/*R*_g_) as functions of xylene concentration (5 to 0.1 ppm). (**c**) Eight repetitive sensing transients to 5 ppm xylene at 300 °C.

The gas-sensing characteristic of the 2Sb-SnO_2_ sensor was further investigated by varying the xylene concentration between 0.1 and 5 ppm at 300 °C, where a concentration-dependent sensing behavior toward xylene was observed (Figure 9a). Based on the gas responses to 0.1–5 ppm xylene of the 2Sb-SnO_2_ sensor, the detection limit was estimated to be 50 ppb from a linear fit of the logarithmic *S*_X_ values, using *R*_a_/*R*_g_ > 1.2 as the criterion (Figure 9b). In addition, the sensor showed reproducible responses over eight repetitive exposures to 5 ppm of xylene 300 °C (Figure 9c). Moreover, Table 2 compares the sensing performance of the 2Sb-SnO_2_ sensor with previously reported xylene sensors. Within comparable operating conditions, the 2Sb-SnO_2_ sensor demonstrates a competitive response while maintaining a significantly lower sensor resistance compared to other reported systems [24,26,27,28,47,48,49], which often exhibit an excessively high resistance value range. Notably, the present sensor achieves a balanced combination of high response and practical resistance (38.5 kΩ), which is advantageous for cost-effective signal measurement and device integration. These findings suggest that 2Sb-SnO_2_ is capable of detecting trace levels of xylene, as a harmful indoor air pollutant.

We further evaluated the sensor performance under a humid atmosphere of 50% relative humidity (RH). Humidity-dependent measurements were performed using 2.5 ppm xylene and ethanol as representative target and interfering gases (Figure S5). The reduced gas concentration compared to dry conditions (5 ppm) originates from unavoidable dilution effects when humid air (50% RH) is introduced into the gas-mixing system. Under humid conditions, both the baseline resistance and the gas response of the 2Sb-SnO_2_ sensor decreased compared to dry-air conditions, which is consistent with the behavior typically observed in metal oxide semiconductor sensors [32,50,51]. Importantly, the sensor continued to exhibit clear selectivity toward xylene over ethanol. These results indicate that, although the resistance and response magnitude are reduced under humid conditions, the proposed sensor remains operable in humid environments, supporting its potential applicability in practical applications.

## 4. Conclusions

In this study, we designed Sb-doped SnO_2_ hollow spheres for highly selective and sensitive xylene detection without increased sensor resistance. The 2Sb-SnO_2_ hollow spheres showed high selectivity (*S*_X_/*S*_E_ = 3.4) and response (*S*_X_ = 24.0) to 5 ppm of xylene at 300 °C. Moreover, Sb doping significantly reduced the sensor resistance by increasing the background charge-carrier concentration through the substitution of Sb^5+^ into Sn^4+^ lattice sites. Note that 2 mol% Sb doping provided significant advantages, including an approximately 200-fold reduction in resistance and enhanced xylene selectivity and response without changing *τ*_res_ and *τ*_recov_. Among the studied samples, 2 mol% Sb doping showed the most favorable trade-offs; however, finer composition screening may further refine the optimum. In addition, the 2Sb-SnO_2_ sensor exhibited a low detection limit of 50 ppb of xylene. These results indicate that Sb addition is an effective strategy for designing low-resistance SnO_2_ sensors. This approach can be widely utilized for designing highly selective and sensitive gas sensors without increasing sensor resistance.

## Figures and Tables

**Table 1:** Sensor resistance in air (*R*_a_) of pure thick SnO_2_ and Sb-doped SnO_2_ sensors.

Sample	Resistance (kΩ)
SnO_2_	7223.8
2Sb-SnO_2_	38.5
5Sb-SnO_2_	23.9
10Sb-SnO_2_	0.8

**Table 2:** Properties of various materials used for xylene sensing reported in the literature and obtained in this study [24,26,27,28,47,48,49].

Material	Conc. [ppm]	Response [*R_a_R_g_*^−1^ − 1, *R_g_R_a_*^−1^ − 1]	Sensor Resistance [kΩ]	Sensor Temp. [°C]	Ref.
Nb-doped NiO hollow spheres	5	1171	5400	350	[26]
Cr-doped NiO hierarchical nanostructures	5	11.6	~200	400	[27]
CuO-ZnO heterostructure-based sensor	5	1.9	~1	100	[28]
Pt-Cr_2_O_3_-WO_3_ nanofiber	10	74.3	~150,000	325	[47]
CuO/WO_3_ hierarchical structure	50	6.4	~328	260	[48]
NiO/NiCr_2_O_4_ nanoparticles	100	66.2	~2700	225	[49]
Au-loaded MoO_3_ hollow spheres	100	22.1	~900,000	250	[24]
2Sb-SnO_2_ hollow spheres	5	24.0	38.5	300	This work

## Data Availability

The data presented in this study are available on request from the corresponding author.

## Supplementary Materials

The following supporting information can be downloaded at: https://www.mdpi.com/article/10.3390/nano16050313/s1, Note S1. Gas selectivity comparison. Note S2. Comparison of response and recovery times. Figure S1. Schematic illustration of (a) ultrasonic spray pyrolysis and (b) sensor fabrication process. Figure S2. Average diameter comparison graph for the pure and *x*Sb-SnO_2_ sensors. Figure S3. XPS survey spectrum of 2Sb-SnO_2_ sensor. Figure S4. (a) Gas-sensing characteristics (at 250–400 °C) and (b) gas response (at 300 °C) of the 1Sb-doped SnO_2_ sensors to 5 ppm of analytes. Figure S5. Gas response (at 300 °C, RH50%) of the 2Sb-doped SnO_2_ sensors to 2.5 ppm of analytes.
