# Supplementary material for: Sb-Doped SnO2 Hollow Spheres for Low-Resistance and Highly Selective Xylene Sensors
Source: Nanomaterials (Basel). 2026 Feb 28;16(5):313. doi: 10.3390/nano16050313 (PMC12985920; doi:10.3390/nano16050313)
Supplement: Supplementary file 1 [file nanomaterials-16-00313-s001.zip › nanomaterials-4147543-supplementary.pdf]

Sb-Doped SnO_2 Hollow Spheres for Low-Resistance and Highly Selective Xylene Sensors

Jung-Hoo Seo,[†] Seong-Young Yoon,[†] Sang-Myeong Lee and Seong-Yong Jeong*

Division of Advanced Materials Engineering, Kongju National University, Cheonan 31080, Chungnam, Republic of Korea.

*Correspondence: syjeong@kongju.ac.kr

Note S1 | Gas selectivity comparison.

Ethanol is a ubiquitous gas produced by alcoholic beverages and cleaning products. Thus, the xylene gas selectivity (S_X/S_E) was calculated based on the ratio of the xylene response to the major interference ethanol response, and the results are plotted as functions of the sensing temperature (Fig. 6).

Note S2 | Comparison of response and recovery times.

The response and recovery kinetics (τ_{res} and τ_{recov}: the times required to reach 90% of the resistance variation when a sensor is exposed to 5 ppm of xylene and ambient air, respectively) of the pure SnO_2 and Sn-SnO_2 sensors were further calculated based on the sensing transients (Fig. 7).

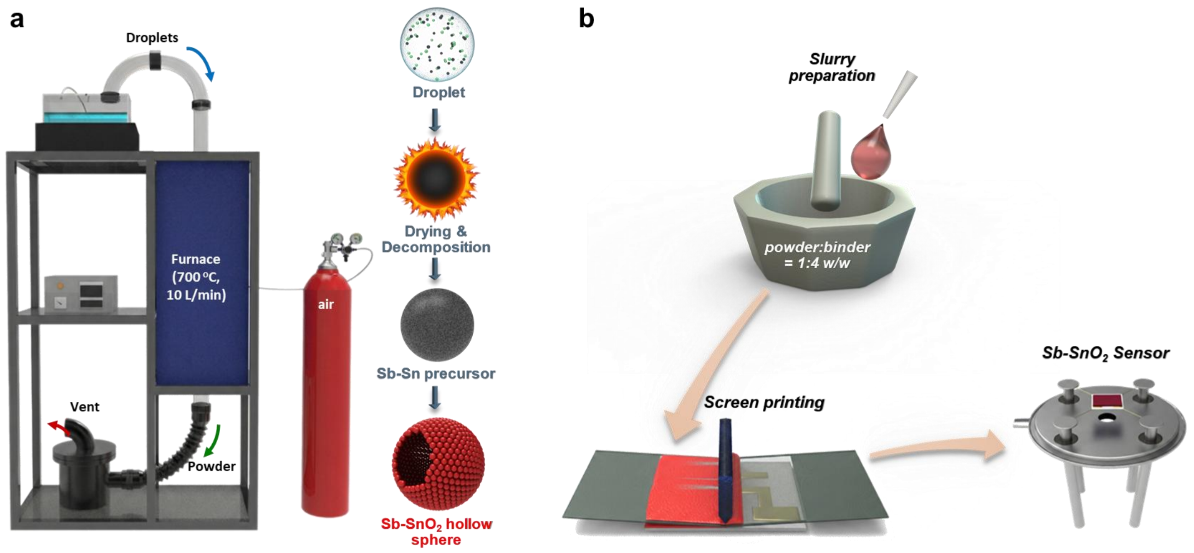

Figure S1. Schematic illustration of (a) ultrasonic spray pyrolysis and (b) sensor fabrication process.

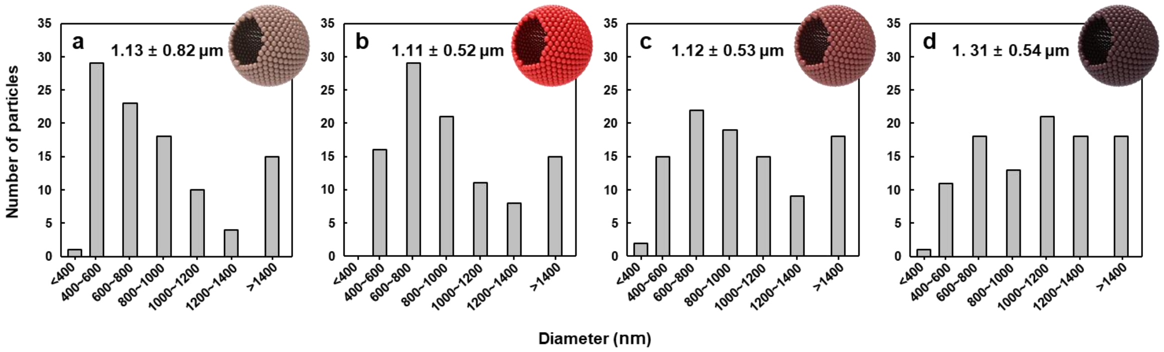

Figure S2. Average diameter comparison graph for the pure and *x*Sb-SnO_2 sensors.

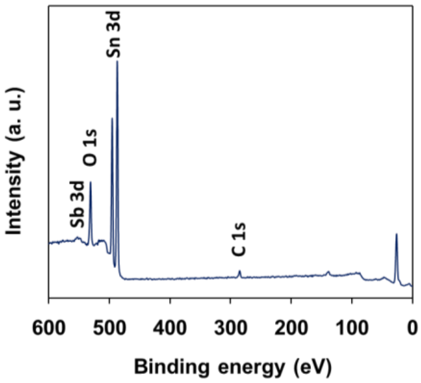

Figure S3. XPS survey spectrum of 2Sb-SnO_2 sensor.

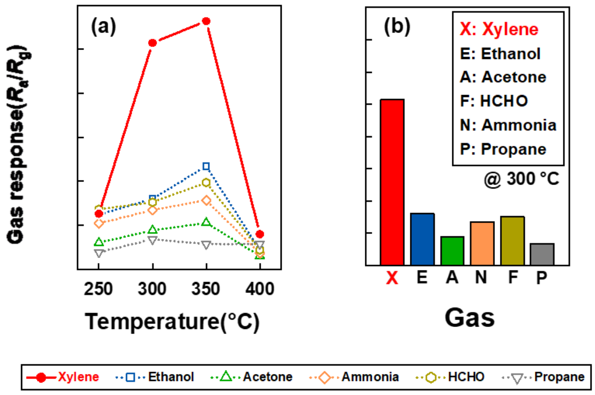

Figure S4. (a) Gas-sensing characteristics (at 250–400 °C) and (b) gas response (at 300 °C) of the 1Sb-doped SnO_2 sensors to 5 ppm of analytes.

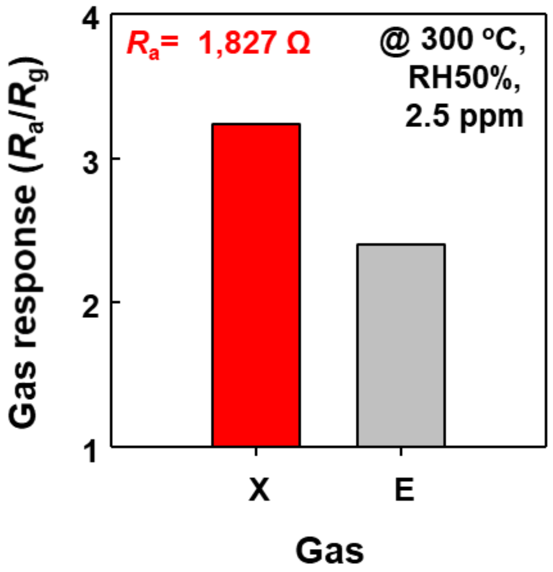

Figure S5. Gas response (at 300 °C, RH50%) of the 2Sb-doped SnO_2 sensors to 2.5 ppm of analytes.
